# The Multiple Dimensions of Gender Stereotypes: A Current Look at Men’s and Women’s Characterizations of Others and Themselves

**DOI:** 10.3389/fpsyg.2019.00011

**Published:** 2019-01-30

**Authors:** Tanja Hentschel, Madeline E. Heilman, Claudia V. Peus

**Affiliations:** ^1^TUM School of Management, Technische Universität München, Munich, Germany; ^2^Amsterdam Business School, University of Amsterdam, Amsterdam, Netherlands; ^3^Department of Psychology, New York University, New York, NY, United States

**Keywords:** gender stereotypes, self-stereotyping, communality, communion, agency, men, women, gender identity

## Abstract

We used a multi-dimensional framework to assess current stereotypes of men and women. Specifically, we sought to determine (1) how men and women are characterized by male and female raters, (2) how men and women characterize themselves, and (3) the degree of convergence between self-characterizations and charcterizations of one’s gender group. In an experimental study, 628 U.S. male and female raters described men, women, or themselves on scales representing multiple dimensions of the two defining features of gender stereotypes, agency and communality: assertiveness, independence, instrumental competence, leadership competence (agency dimensions), and concern for others, sociability and emotional sensitivity (communality dimensions). Results indicated that stereotypes about communality persist and were equally prevalent for male and female raters, but agency characterizations were more complex. Male raters generally descibed women as being less agentic than men and as less agentic than female raters described them. However, female raters differentiated among agency dimensions and described women as less assertive than men but as equally independent and leadership competent. Both male and female raters rated men and women equally high on instrumental competence. Gender stereotypes were also evident in self-characterizations, with female raters rating themselves as less agentic than male raters and male raters rating themselves as less communal than female raters, although there were exceptions (no differences in instrumental competence, independence, and sociability self-ratings for men and women). Comparisons of self-ratings and ratings of men and women in general indicated that women tended to characterize themselves in more stereotypic terms – as less assertive and less competent in leadership – than they characterized others in their gender group. Men, in contrast, characterized themselves in less stereotypic terms – as more communal. Overall, our results show that a focus on facets of agency and communality can provide deeper insights about stereotype content than a focus on overall agency and communality.

## Introduction

There is no question that a great deal of progress has been made toward gender equality, and this progress is particularly evident in the workplace. There also is no question that the goal of full gender equality has not yet been achieved – not in pay (AAUW, 2016) or position level (Catalyst, 2016). In a recent interview study with female managers the majority of barriers for women’s advancement that were identified were consequences of gender stereotypes (Peus et al., 2015). There is a long history of research in psychology that corroborates this finding (for reviews see Eagly and Sczesny, 2009; Heilman, 2012). These investigations support the idea that gender stereotypes can be impediments to women’s career advancement, promoting both gender bias in employment decisions and women’s self-limiting behavior (Heilman, 1983).

This study is designed to investigate the current state of gender stereotypes about men and women using a multi-dimensional framework. Much of the original research on the content of gender stereotypes was conducted several decades ago (e.g., Rosenkrantz et al., 1968), and more recent research findings are inconsistent, some suggesting that there has been a change in traditional gender stereotypes (e.g., Duehr and Bono, 2006) and others suggesting there has not (e.g., Haines et al., 2016). Measures of stereotyping in these studies tend to differ, all operationalizing the constructs of agency and communality, the two defining features of gender stereotypes (Abele et al., 2008), but in different ways. We propose that the conflict in findings may derive in part from the focus on different facets of these constructs in different studies. Thus, we seek to obtain a more complete picture of the specific content of today’s gender stereotypes by treating agency and communality, as multi-dimensioned constructs.

Gender stereotypes often are internalized by men and women, and we therefore focus both on how men and women are seen by others and how they see themselves with respect to stereotyped attributes. We also plan to compare and contrast charcterizations of men or women as a group with charcterizations of self, something not typically possible because these two types of characterizations are rarely measured in the same study. In sum, we have multiple objectives: We aim to develop a multi-dimensional framework for assessing current conceptions of men’s and women’s characteristics and then use it to consider how men and women are seen by male and female others, how men and women see themselves, and how these perceptions of self and others in their gender group coincide or differ. In doing so, we hope to demonstrate the benefits of viewing agency and communality as multidimensional constructs in the study of gender stereotypes.

### Gender Stereotypes

Gender stereotypes are generalizations about what men and women are like, and there typically is a great deal of consensus about them. According to social role theory, gender stereotypes derive from the discrepant distribution of men and women into social roles both in the home and at work (Eagly, 1987, 1997; Koenig and Eagly, 2014). There has long been a gendered division of labor, and it has existed both in foraging societies and in more socioeconomically complex societies (Wood and Eagly, 2012). In the domestic sphere women have performed the majority of routine domestic work and played the major caretaker role. In the workplace, women have tended to be employed in people-oriented, service occupations rather than things-oriented, competitive occupations, which have traditionally been occupied by men (e.g., Lippa et al., 2014). This contrasting distribution of men and women into social roles, and the inferences it prompts about what women and men are like, give rise to gender stereotypical conceptions (Koenig and Eagly, 2014).

Accordingly, men are characterized as more *agentic* than women, taking charge and being in control, and women are characterized as more *communal* than men, being attuned to others and building relationships (e.g., Broverman et al., 1972; Eagly and Steffen, 1984). These two concepts were first introduced by Bakan (1966) as fundamental motivators of human behavior. During the last decades, agency (also referred to as “masculinity,” “instrumentality” or “competence”) and communality (also referred to as “communion,” “femininity,” “expressiveness,” or “warmth”) have consistently been the focus of research (e.g., Spence and Buckner, 2000; Fiske et al., 2007; Cuddy et al., 2008; Abele and Wojciszke, 2014). These dual tenets of social perception have been considered fundamental to gender stereotypes.

Stereotypes can serve an adaptive function allowing people to categorize and simplify what they observe and to make predictions about others (e.g., Devine and Sharp, 2009; Fiske and Taylor, 2013). However, stereotypes also can induce faulty assessments of people – i.e., assessments based on generalization from beliefs about a group that do not correspond to a person’s unique qualities. These faulty assessments can negatively or positively affect expectations about performance, and bias consequent decisions that impact opportunities and work outcomes for both men and women (e.g., Heilman, 2012; Heilman et al., 2015; Hentschel et al., 2018). Stereotypes about gender are especially influential because gender is an aspect of a person that is readily noticed and remembered (Fiske et al., 1991). In other words, gender is a commonly occurring cue for stereotypic thinking (Blair and Banaji, 1996).

Gender stereotypes are used not only to characterize others but also to characterize oneself (Bem, 1974). The process of self-stereotyping can influence people’s identities in stereotype-congruent directions. Stereotyped characteristics can thereby be internalized and become part of a person’s gender identity – a critical aspect of the self-concept (Ruble and Martin, 1998; Wood and Eagly, 2015). Young boys and girls learn about gender stereotypes from their immediate environment and the media, and they learn how to behave in gender-appropriate ways (Deaux and LaFrance, 1998). These socialization experiences no doubt continue to exert influence later in life and, indeed, research has shown that men’s and women’s self-characterizations differ in ways that are stereotype-consistent (Bem, 1974; Spence and Buckner, 2000).

#### Measurement of Gender Stereotypes

Gender stereotypes, and their defining features of agency and communality, have been measured in a variety of ways (Kite et al., 2008). Researchers have investigated people’s stereotypical assumptions about how men and women differ in terms of, for example, ascribed traits (e.g., Williams and Best, 1990), role behaviors (e.g., Haines et al., 2016), occupations (e.g., Deaux and Lewis, 1984), or emotions (e.g., Plant et al., 2000). Researchers also have distinguished personality, physical, and cognitive components of gender stereotypes (Diekman and Eagly, 2000). In addition, they have investigated how men’ and women’s self-characterizations differ in stereotype-consistent ways (Spence and Buckner, 2000).

Today, the most common measures of gender stereotypes involve traits and attributes. Explicit measures of stereotyping entail responses to questionnaires asking for descriptions of men or women using Likert or bi-polar adjective scales (e.g., Kite et al., 2008; Haines et al., 2016), or asking for beliefs about the percentage of men and women possessing certain traits and attributes (e.g., McCauley and Stitt, 1978). Gender stereotypes have also been studied using implicit measures, using reaction time to measure associations between a gender group and a stereotyped trait or attribute (e.g., Greenwald and Banaji, 1995). Although implicit measures are used widely in some areas of research, our focus in the research reported here builds on the longstanding tradition of measuring gender stereotypes directly through the use of explicit measures.

### Contemporary Gender Stereotypes

Researchers often argue that stereotypes are tenacious; they tend to have a self-perpetuating quality that is sustained by cognitive distortion (Hilton and von Hippel, 1996; Heilman, 2012). However, stereotype maintenance is not only a product of the inflexibility of people’s beliefs but also a consequence of the societal roles women and men enact (Eagly and Steffen, 1984; Koenig and Eagly, 2014). Therefore, the persistence of traditional gender stereotypes is fueled by skewed gender distribution into social roles. If there have been recent advances toward gender equality in workforce participation and the rigid representation of women and men in long-established gender roles has eased, then might the content of gender stereotypes have evolved to reflect this change?

The answer to this question is not straightforward; the degree to which there has been a true shift in social roles is unclear. On the one hand, there are more women in the workforce than ever before. In 1967, 36% of U.S. households with married couples were made up of a male provider working outside the home and a female caregiver working inside the home, but now only 19% of U.S. households concur with this division (Bureau of Labor Statistics, 2017). Moreover, women increasingly pursue traditionally male careers, and there are more women in roles of power and authority. For example, today women hold almost 40% of management positions in the United States (Bureau of Labor Statistics, 2017). In addition, more men are taking on a family’s main caretaker role (Ladge et al., 2015). Though families with only the mother working are still rare (5% in 2016 compared to 2% in 1970), the average number of hours fathers spent on child care per week increased from 2.5 to 8 h in the last 40 years (Pew Research Center, 2018). In addition, the majority of fathers perceive parenting as extremely important to their identity (Pew Research Center, 2018).

On the other hand, role segregation, while somewhat abated, has by no means been eliminated. Despite their increased numbers in the labor force, women still are concentrated in occupations that are perceived to require communal, but not agentic attributes. For example, the three most common occupations for women in the U.S. involve care for others (elementary and middle school teacher, registered nurse, and secretary and administrative assistant; U.S. Department of Labor, 2015), while men more than women tend to work in occupations requiring agentic attributes (e.g., senior management positions, construction, or engineering; Bureau of Labor Statistics, 2016b). Sociological research shows that women are underrepresented in occupations that are highly competitive, inflexible, and require high levels of physical skill, while they are overrepresented in occupations that place emphasis on social contributions and require interpersonal skills (Cortes and Pan, 2017). Moreover, though men’s home and family responsibilities have increased, women continue to perform a disproportionate amount of domestic work (Bureau of Labor Statistics, 2016a), have greater childcare responsibilities (Craig and Mullan, 2010; Kan et al., 2011), and continue to be expected to do so (Park et al., 2008).

Thus, there is reason both to expect traditional gender stereotypes to dominate current conceptions of women and men, and to expect them to not. Relevant research findings are conflicting. For example, a large investigation found that over time managers have come to perceive women as more agentic (Duehr and Bono, 2006). However, other investigations have found gender stereotypes to have changed little over time (Heilman et al., 1989) or even to have intensified (Lueptow et al., 2001). A recent study replicating work done more than 30 years ago found minimal change, with men and women still described very differently from one another and in line with traditional stereotyped conceptions (Haines et al., 2016).

There also have been conflicting findings concerning self-charcterizations, especially in women’s self-views of their agency. Findings by Abele (2003) suggest that self-perceived agency increases with career success. Indeed, there has been indication that women’s self-perceived deficit in agency has abated over time (Twenge, 1997) or that it has abated in some respects but not others (Spence and Buckner, 2000). However, a recent meta-analysis has found that whereas women’s self-perceptions of communality have decreased over time, their self-perceptions of agency have remained stable since the 1990s (Donnelly and Twenge, 2017). Yet another study found almost no change in men’s and women’s self-characterizations of their agency and communality since the 1970s (Powell and Butterfield, 2015).

There are many possible explanations for these conflicting results. A compelling one concerns the conceptualization of the agency and communality constructs and the resulting difference in the traits and behaviors used to measure them. In much of the gender stereotypes literature, agency and communality have been loosely used to denote a set of varied attributes, and different studies have operationalized agency and communality in different ways. We propose that agency and communality are not unitary constructs but rather are comprised of multiple dimensions, each distinguishable from one another. We also propose that considering these dimensions separately will enhance the clarity of our understanding of current differences in the characterization of women and men, and provide a more definitive picture of gender stereotypes today.

### Dimensions of Communality and Agency

There has been great variety in how the agency construct has been operationalized, and the specific terms used to measure agency often differ from study to study (e.g., McAdams et al., 1996; Rudman and Glick, 2001; Abele et al., 2008; Schaumberg and Flynn, 2017). Furthermore, distinctions between elements of agency have been identified: In a number of studies competence has been shown to be distinct from agency as a separate factor (Carrier et al., 2014; Koenig and Eagly, 2014; Abele et al., 2016; Rosette et al., 2016), and in others, the agency construct has been subdivided into self-reliance and dominance (Schaumberg and Flynn, 2017). There also has been great variety in how the communality construct has been operationalized (Hoffman and Hurst, 1990; Fiske et al., 2007; Abele et al., 2008; Brosi et al., 2016; Hentschel et al., 2018). Although there have been few efforts to pinpoint specific components of communality, recent work focused on self-judgments in cross-cultural contexts has subdivided it into facets of warmth and morality (Abele et al., 2016).

The multiplicity of items used to represent agency and communality in research studies involving stereotyping is highly suggestive that agentic and communal content can be decomposed into different facets. In this research we seek to distinguish dimensions underlying both the agency and the communality constructs. Our aim is to lend further credence to the idea that the fundamental constructs of agency and communality are multifaceted, and to supply researchers with dimensions of each that may be useful for study of stereotype evaluation and change.

While we are proposing that agency and communality can be broken down into components, we are not claiming that the use of these overarching constructs in earlier research has been an error. In the vast majority of studies in which communality or agency has been measured the scale reliabilities have been high and the items highly correlated. However, internal consistency does not necessarily indicate that the individual items included are unidimensional (Schmitt, 1996; Sijtsma, 2008), or that the entirety of the construct is being captured in a particular measure. Moreover, there are multiple meanings included in these constructs as they have been discussed and operationalized in gender research. Therefore, we propose that breaking them down into separate dimensions will provide finer distinctions about contemporary characterizations of men and women.

### Perceiver Sex

Findings often demonstrate that male and female raters are equally likely to characterize women and men in stereotypic terms (Heilman, 2001, 2012). This suggests that stereotypes outweigh the effects of evaluators’ gender identities and, because men and women live in the same world, they see the world similarly. However, the steady shift of women’s societal roles and its different implications for men and women may affect the degree to which men and women adhere to traditional gender stereotypes.

On the face of it, one would expect women to hold traditional gender stereotypes less than men. The increase of women in the workforce generally, and particularly in domains typically reserved for men, is likely to be very salient to women. Such changes have distinct implications for them – implications that can impact their expectations, aspirations, and actual experiences. As a result, women may be more attentive than men to shifts in workplace and domestic roles, and more accepting of these roles as the new status quo. They consequently may be more amenable to incorporating updated gender roles into their understanding of the world, diminishing stereotypic beliefs.

Unlike women, who may be likely to embrace recent societal changes, men may be prone to reject or dismiss them. The same societal changes that present new opportunities for women can present threats to men, who may see themselves as losing their rightful place in the social order (see also Sidanius and Pratto, 1999; Knowles and Lowery, 2012). Thus, men may be less willing to accept modern-day changes in social roles or to see these changes as definitive. There may be little impetus for them to relinquish stereotypic beliefs and much impetus for them to retain these beliefs. If this is the case, then men would be expected to adhere more vigorously to traditional gender stereotypes than women.

### Self-Stereotyping Versus Stereotyping of One’s Gender Group

Although gender stereotypes impact charcterizations of both self and others, there may be a difference in the degree to which stereotypes dominate in self- and other-characterizations. That is, women may see themselves differently than they see women in general and men may see themselves differently than they see men in general; although they hold stereotypes about their gender groups, they may not apply them to themselves. Indeed, attribution theory (Jones and Nisbett, 1987), which suggests that people are more prone to attribute behavior to stable personality traits when viewing someone else than when viewing oneself, gives reason to argue that stereotypes are more likely to be used when characterizing others in one’s gender group than when characterizing oneself. A similar case can be made for construal level theory (Trope and Liberman, 2010), which suggests that psychological distance promotes abstraction rather than attention to individuating information. Moreover, the impact of societal changes that affect adherence to gender stereotypes is apt to have greater immediacy and personal impact for self, and therefore be more reflected in self-characterizations than in characterizations of others.

Some studies have compared the use of stereotypes in characterizing self and others. In an early study (Rosenkrantz et al., 1968), each participating student was asked to rate men, women, and self on a number of characteristics. The researchers found that self-characterizations of men and women showed less evidence of stereotypes than characterizations of others. Similar results were found in studies on accuracy of stereotyping (Martin, 1987; Allen, 1995). Using instrumenal (i.e., agentic) and expressive (i.e., communal) attributes from the BSRI and PAQ scales, Spence and Buckner (2000) found very little relation between stereotypes about others and self-characterizations.

There is reason to think that some dimensions of gender stereotypes are more likely than others to be differentially subscribed to when characterizing self than when characterizing others. For example, there is a tendency to boost self-esteem and adopt descriptors that are self-enhancing when describing oneself (Swann, 1990), and this may have conseqences whether these descriptors are consistent or inconsistent with gender stereotypes. If this is so, gender may be an important factor; there are likely particular aspects of gender stereotypes that are more (or less) acceptable to women and men, affecting the degree to which they are reflected in men’s and women’s self-descriptions as compared to their description of their gender group. However, there also is reason to believe that individuals will embrace positive stereotypes and reject negative stereotypes as descriptive not only of themselves but also of their close in-groups (Biernat et al., 1996), suggesting that there will be little difference between characterizations of oneself and one’s gender group. Therefore, to obtain a full picture of the current state of gender stereotypes and their impact on perceptions, we believe it important to compare self-characterizations and characterizations of one’s gender group on specific dimensions of gender stereotypes.

### Overview of the Research

In this study, we develop a multidimensional framework for measuring different elements of agency and communality to provide an assessment of contemporary gender stereotypes and their impact on charcterizations about others and self. Using the multidimensional framework, we sought to determine (1) if men and women differ in their gender stereotypes; (2) if men and women differ in their self-characterizations; and (3) if men’s and women’s self-characterizations differ from their characterizations of their gender groups. In each instance we compare the results using the traditional unidimensional framework for measuring agency and communality with the results using the newly formulated multidimensional framework.

## Materials and Methods

### Participants

Six hundred and twenty-nine participants (61% female, all U.S. residents) were recruited online via Amazon Mechanical Turk (MTurk), providing a more representative sample of the U.S. population than student samples. MTurk samples tend to be slightly more diverse than and similarly reliable as other types of internet samples used in psychological research (Paolacci et al., 2010; Buhrmester et al., 2011), but nonetheless are convenience samples rather than true representative samples based on demographic data (see e.g., Pew Research Center, 2017). In our sample, ages ranged from 19 to 83, with a mean age of 34.5 years (*SD* = 13.1). In addition, education ranged from those who had not attended college (17%), had some college education (33%), had graduated from college (37%), to those who had graduate degrees (13%). 77.6% self-identified as White, 8.4% Asian, 7.0% African American, 4.8% Hispanic, and 2.2% other.^1^ The survey link was visible only to U.S. residents who had a greater than 95% acceptance rate of previous MTurk work, an indication that their earlier work had been handled responsibly. In addition, we included a question asking participants to indicate whether they filled out the questionnaire honestly (we assured them that their answer on this question would not have any consequences for their payment). One person indicated that he had not filled out the survey honestly and was excluded from the analyses.

### Design

We conducted an experiment with two independent variables: rater gender (male or female) and target group (men in general, women in general, or self). The target group manipulation was randomly assigned to male and female raters. Subsets of this overall design were used to address our specific research questions.

### Procedure

Participants were told that we were interested in people perception, and they were asked either to rate men in general (*N* = 215) women in general (*N* = 208) or themselves (*N* = 205) on an attribute inventory representing various dimensions of agency and communality^2^. The attributes were presented in differing orders to participants, randomized by the survey tool we used. Ratings were made using a 7-point scale with responses ranging from 1 (“not at all”) to 7 (“very much”).

### Scale Construction

Using an inductive procedure, scale development proceeded in four steps. In the first step, we identified a set of 74 attributes, representative of how agency and communality have been measured by researchers in the past (consisting of adjectives, traits, and descriptors; see Appendix Tables A, B for the full list). The attributes were chosen from earlier investigations of gender stereotypes, including those of Broverman et al. (1972), Schein (1973), Spence and Helmreich (1978), Heilman et al. (1995), Fiske et al. (1999), Diekman and Eagly (2000), and Oswald and Lindstedt (2006). They were selected to represent a broad array of agentic and communal attributes with a minimal amount of redundancy.

In the second step, three judges (the first two authors and another independent researcher) sorted the descriptive attributes into categories based on their conceptual similarity. The total set of attributes measured was included in the sorting task, and there was no limit placed on the number of categories to be created and no requirements for the number of attributes to be included within each created category. Specifically, the instructions were to use as many categories as needed to sort the attributes into conceptually distinct groupings. The sorting results were then discussed by the judges and two additional researchers. During the discussion, agreement was reached about the number of categories necessary to best capture the distinct dimensions of the sorted attributes. Attributes for which no consensus was reached about category placement were omitted. Then decisions were made about how each of the categories should be labeled. Seven categories were identified, four of which represented dimensions of agency – instrumental competence, leadership competence, assertiveness, independence – and three of which represented dimensions of communality – concern for others, sociability, emotional sensitivity.

In the third step, we had a different set of three independent judges (all graduate students in a psychology program) do a sorting of the retained attributes into the labeled categories. This was done to make sure that their sorting conformed to the identified categories; items that were misclassified by any of the judges were eliminated from the item set.

Finally, in a fourth step, we used confirmatory factor analysis procedures to further hone our categories. Following standard procedures on increasing model fit (e.g., Byrne, 2010), we eliminated all items that showed a low fit to the created categories. We later conducted a conclusive confirmatory factor analysis, for which the results are reported in the next section.

As a result of these steps, we created seven scales, each composed of the attributes remaining in one of the seven designated categories. The scales ranged from 3 to 4 items, the coefficient alphas all surpassed 0.75, and all corrected item-scale correlations surpassed 0.40 (Field, 2006). Table 1 presents the attributes comprising each of the scales as well as the Cronbach alphas and corrected-item-scale correlations.

**Table 1:** Dimension scales, scale items, and reliability information.

Agency dimensions	Corrected item-scale correlation	Communality dimensions	Corrected item-total correlation
**Instrumental Competence (α = 0.88)**		**Concern for Others (α = 0.91)**	
Competent	0.74	Understanding	0.75
Effective	0.79	Kind	0.79
Productive	0.78	Compassionate	0.82
Task-Oriented	0.67	Sympathetic	0.80
**Leadership Competence (α = 0.80)**		**Sociability (α = 0.77)**	
Leadership Ability	0.71	Communicative	0.62
Achievement-Oriented	0.62	Collaborative	0.58
Skilled In Business Matters	0.62	Relationship-oriented	0.52
**Assertiveness (α = 0.80)**		Likeable	0.60
Dominant	0.62	**Emotional Sensitivity (α = 0.75)**	
Bold	0.56	Emotional	0.59
Assertive	0.66	Intuitive	0.47
Competitive	0.60	Sentimental	0.68
**Independence (α = 0.82)**			
Independent	0.72		
Desires Responsibility	0.56		
Emotionally Stable	0.60		
Self-Reliant	0.69		


The four scales composed of agentic attributes and denoting dimensions of agency were: instrumental competence, leadership competence, assertiveness, and independence. Thus, the sorting process not only distinguished between competence and other elements of agency (as has been suggested by others like Carrier et al., 2014), but further decomposed the non-competence elements of agency into dimensions of assertiveness and independence. Assertiveness concerns acting on the world and taking charge. Independence connotes self-reliance and acting on one’s own, free of the influence of others. Furthermore, competence was subdivided into two separate dimensions – one focused on performance execution (instrumental competence), and the other focused on capability to perform as a leader (leadership competence). Both leadership competence and assertiveness imply high social power whereas instrumental competence and independence are not typically associated with power relations.

The three scales composed of communal attributes and denoting dimensions of communality were: concern for others, sociability, and emotional sensitivity. Concern for others and sociability both entail a focus on others, but the former involves a one-way relationship of giving and nurturance while the latter involves a transactional relationship focused on relationship building. Emotional sensitivity implies an orientation that focuses on feelings as an antecedent or consequence of interactions with others.

#### Confirmatory Factor Analysis

We conducted a confirmatory factor analysis using the R package lavaan (Rosseel, 2012) to test the factor structure of the four final agency scales and the three final communality scales. Results revealed that for agency, the theoretically assumed four-factor model (i.e., instrumental competence, leadership competence, assertiveness, and independence as first-order factors) provided adequate fit (χ^2^ = 370.224, df = 84, *p* < 0.001, χ^2^/df = 4.41, CFI = 0.947, RMSEA = 0.076, SRMR = 0.045) and also was more suitable than a one-factor model in which all agency items loaded on a single factor (χ^2^ = 813.318, df = 90, *p* < 0.001, χ^2^/df = 9.04, CFI = 0.866, RMSEA = 0.116, SRMR = 0.068). A comparison of the two models showed that the four-factor agency model differed significantly from the one-factor model and was thus preferable (Δχ^2^ = 443.09, df = 6, *p* < 0.001). Similarly, for communality the theoretically posited three-factor model (i.e., concern for others, sociability, and emotional sensitivity as first-order factors) provided acceptable fit (χ^2^ = 326.000, df = 41, *p* < 0.001, χ^2^/df = 7.95, CFI = 0.931, RMSEA = 0.108, SRMR = 0.048)^3^ and was more suitable than the one-factor model in which all communality items loaded on a single factor (χ^2^ = 359.803, df = 44, *p* < 0.001, χ^2^/df = 7.95, CFI = 0.924, RMSEA = 0.110, SRMR = 0.048). A comparison of the two models showed that the three-factor communality model differed significantly from the one-factor model and was therefore preferable (Δχ^2^ = 33.80, df = 3, *p* < 0.001). Overall, these results indicated that even though there were high correlations among the agency scales and also among the communality scales (as we would expect given our idea that in each case the multiple scales are part of the same construct; see Table 2), the four scales for agency and the three scales for communality represent different dimensions of these constructs.

**Table 2:** Descriptive statistics and intercorrelations of agentic and communal dimension scales.

	A				B		
		
Dimension Scales	1	2	3	4	5	6	7
**(A) Agentic Dimensions**							
(1) Instrumental Competence	–						
(2) Leadership Competence	0.77^∗∗∗^	–					
(3) Assertiveness	0.52^∗∗∗^	0.69^∗∗∗^	–				
(4) Independence	0.81^∗∗∗^	0.78^∗∗∗^	0.58^∗∗∗^	–			
**(B) Communal Dimensions**							
(5) Concern for Others	0.63^∗∗∗^	0.38^∗∗∗^	0.13^∗∗^	0.50^∗∗∗^	–		
(6) Sociability	0.70^∗∗∗^	0.53^∗∗∗^	0.29^∗∗∗^	0.57^∗∗∗^	0.80^∗∗∗^	–	
(7) Emotional Sensitivity	0.44^∗∗∗^	0.21^∗∗∗^	0.02	0.27^∗∗∗^	0.77^∗∗∗^	0.72^∗∗∗^	–


*^∗∗^*p* < 0.01; ^∗∗∗^*p* < 0.001.*

### Overall Measures

To provide a point of comparison for our multi-dimensional framework, we also determined scales for overall agency and overall communality. In other words, the 15 agency items were combined into one overall agency scale (α = 0.93) and the 11 communality items were combined into one overall communality scale (α = 0.93).

## Results

### Preliminary Analyses: Rater Age and Education Level

Because of potential consequences of raters’ age and education level on the use of gender stereotypes (younger and more educated people might be less likely to adhere to them), we conducted initial analyses to identify their independent and interactive effects. We did not have the opportunity to do the same for race because our subsamples of Asian, African American, and Hispanic participants were not large enough. To determine whether there were differences in the pattern of responses depending upon the age of the rater, we chose the age of 40 as a midlife indicator, divided our sample into two age groups (39 years and younger, 40 years and older), and included age as an additional independent variable in our analyses. Results indicated no main effects or interactions involving age in the ANOVAs conducted. We also divided our sample into two education level groups (those who had graduated from college or had advanced degrees and those who had not graduated from college), and included educational level as an additional independent variable in our analyses. We found no main effects or interactions involving educational level in the ANOVAs. As a consequence we combined data from both younger and older participants and from those who were and were not college educated in the analyses reported below.

### Main Analyses

To address our research questions, we conducted a series of ANOVAs on subsets of our participant sample. For each question, we first conducted ANOVAs on the overall agency scale and the overall communality scale. Then, to determine whether the results differed for different agency and communality dimensions, we conducted mixed-model ANOVAs that included either agency dimension (instrumental competence, leadership competence, assertiveness, independence) or communality dimension as a within-subjects factor (concern for others, sociability, and emotional sensitivity). Fisher’s least significant difference (LSD) method was used to test the question-relevant planned comparisons.

### Do Men and Women Differ in Their Gender Stereotypes?

We used a 2 × 2 ANOVA, with rater gender (male, female) and target group (men in genereal, women in general) to assess differences in men’s and women’s gender stereotypes. We first analyzed the overall agency and communality ratings, and then conducted a 2 × 2 × 4 mixed-model ANOVA including the agency dimensions, and a 2 × 2 × 3 mixed-model ANOVA including the communality dimensions. The mixed-model ANOVA results are presented in Table 3. We followed up with LSD comparisons (see Table 4).

**Table 3:** Results of 2 × 2 × 4 Agency ANOVA and 2 × 2 × 3 Communality ANOVA for stereotype ratings.

	2 × 2 × 4 Agency ANOVA	2 × 2 × 3 Communality ANOVA
Rater Gender Main Effect	*F*(1,418) = 15.55, *p* < 0.001, $\eta_{p}^{2}$ = 0.04	*F*(1,418) = 2.26, *p* = 0.133, $\eta_{p}^{2}$ = 0.01
Target Group Main Effect	*F*(1,418) = 5.51, *p* = 0.019, $\eta_{p}^{2}$ = 0.01	*F*(1,418) = 93.10, *p* < 0.001, $\eta_{p}^{2}$ = 0.18
Dimensions Main Effect	*F*(3,1131) = 9.49, *p* < 0.001, $\eta_{p}^{2}$ = 0.02	*F*(2,830) = 2.81, *p* = 0.061, $\eta_{p}^{2}$ = 0.01
Rater Gender ^∗^ Target Group	*F*(1,418) = 2.31, *p* = 0.129, $\eta_{p}^{2}$ = 0.01	*F*(1,418) = 2.26, *p* = 0.133, $\eta_{p}^{2}$ = 0.01
Dimensions ^∗^ Rater Gender	*F*(3,1131) = 1.71, *p* = 0.169, $\eta_{p}^{2}$ = 0.00	*F*(2,830) = 3.95, *p* = 0.020, $\eta_{p}^{2}$ = 0.01
Dimensions ^∗^ Target Group	*F*(3,1131) = 23.65, *p* < 0.001, $\eta_{p}^{2}$ = 0.05	*F*(2,830) = 16.69, *p* < 0.001, $\eta_{p}^{2}$ = 0.04
Dimensions ^∗^ Rater Gender ^∗^ Target Group	*F*(3,1131) = 1.83, *p* = 0.145, $\eta_{p}^{2}$ = 0.00	*F*(2,830) = 6.68, *p* = 0.001, $\eta_{p}^{2}$ = 0.02


*Results are displayed for a 2 (rater gender: male, female) × 2 (target group: men in general, women in general) × 4 (agency dimension: instrumental competence, leadership competence, assertiveness, independence) ANOVA and a 2 (rater gender: male, female) × 2 (target group: men in general, women in general) × 3 (communality dimension: concern for others, sociability, emotional sensitivity) ANOVA.*

**Table 4:** Means, standard deviations, and LSD results of stereotype ratings.

	Mean Values				LSD Comparisons			
		
	Male Raters		Female Raters		Men in General versus Women in General Rated by		Male Raters versus Female Raters Rating	
				
	Men in General	Women in General	Men in General	Women in General	Male Raters	Female Raters	Men in General	Women in General
**Overall Agency**	4.58 (1.26)	4.15 (1.11)	4.85 (1.15)	4.75 (0.97)	*p* = 0.013	*p* = 0.512	*p* = 0.097	*p* < 0.001
Instrumental Competence	4.41 (1.29)	4.46 (1.32)	4.75 (1.18)	4.94 (1.24)	*p* = 0.805	*p* = 0.216	*p* = 0.058	*p* = 0.006
Leadership Competence	4.64 (1.38)	4.20 (1.25)	5.01 (1.29)	4.93 (1.13)	*p* = 0.024	*p* = 0.620	*p* = 0.040	*p* < 0.001
Assertiveness	4.73 (1.40)	3.99 (1.17)	4.94 (1.30)	4.50 (0.98)	*p* < 0.001	*p* = 0.004	*p* = 0.223	*p* = 0.003
Independence	4.56 (1.31)	3.98 (1.30)	4.73 (1.20)	4.69 (1.11)	*p* = 0.002	*p* = 0.776	*p* = 0.333	*p* < 0.001
**Overall Communality**	4.01 (0.89)	4.86 (1.26)	4.04 (0.73)	5.17 (1.28)	*p* < 0.001	*p* < 0.001	*p* = 0.851	*p* = 0.036
Concern for Others	3.97 (0.95)	4.83 (1.40)	4.19 (0.96)	5.16 (1.38)	*p* < 0.001	*p* < 0.001	*p* = 0.210	*p* = 0.048
Sociability	4.09 (1.02)	4.85 (1.24)	4.17 (0.82)	5.10 (1.28)	*p* < 0.001	*p* < 0.001	*p* = 0.571	*p* = 0.102
Emotional Sensitivity	3.96 (0.94)	4.92 (1.41)	3.66 (1.04)	5.29 (1.37)	*p* < 0.001	*p* < 0.001	*p* = 0.081	*p* = 0.029


*Means (and standard deviations) of male and female raters rating men in general and women in general for all scales. Ratings were given on a 7-point scale from 1 “not at all” to 7 “very much”. LSD comparisons are presented for (1) rating differences of men in general versus women in general for male raters and for female raters, and (2) rater gender differences in characterizations of men in general and women in general.*

#### Agency

The 2 × 2 ANOVA results for the overall agency ratings indicated a main effect for both rater gender, *F*(1,418) = 15.10, *p* < 0.001, $\eta_{p}^{2}$ = 0.04, and target group, *F*(1,418) = 5.52, *p* = 0.019, $\eta_{p}^{2}$ = 0.01. The results of the 2 × 2 × 4 mixed-model ANOVA, including the four agency dimensions as a within-subject factor, repeated the main effects for rater gender and target group and also indicated a main effect for agency dimension and an interaction between agency dimension and target group (see Table 3), suggesting that there were differences in ratings depending on the agency dimension.

##### Differences in ratings of men in general and women in general

**FIGURE 1:**
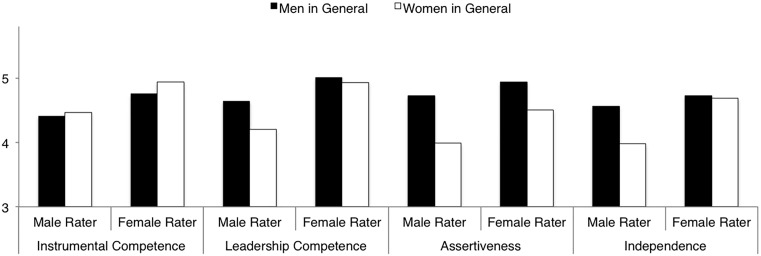
Ratings of agency dimensions (instrumental competence, leadership competence, assertiveness, independence) of men in general and women in general by male and female raters.

LSD comparisons (see Table 4) of the overall agency ratings indicated that male raters rated women in general as lower in overall agency than men in general. They further indicated that female raters rated women in general and men in general as equally agentic. LSD comparisons of the individual agency scales indicated that this result held true for most of the agency dimensions. With the exception of the instrumental competence dimension (on which there were no differences in ratings of women and men in general whether the rater was male or female), male raters rated women in general lower than men in general on the agency dimensions (leaderhip competence, assertiveness, and independence). In contrast to the ratings of male raters but in line with the overall agency result, female raters rated women in general no differently than they rated men in general in leadership competence and independence. Yet, in contrast to the results of the overall agency ratings, female raters differentiated between women and men in ratings of assertiveness. That is, much like male raters, female raters rated women in general as less assertive than men in general. Figure 1 displays the results for the agency dimensions.

##### Rater gender differences in target group characterizations

Additional LSD comparisons (again see Table 4) lent further insight into the source of the gender discrepancy in the comparative ratings of women and men in general. Comparisons of the overall agency ratings indicated that ratings of men in general did not differ as a result of rater gender, but women in general were rated lower by male as compared to female raters. LSD comparisons of the agency dimensions were in line with the overall agency result in ratings of women in general – they were rated lower by male raters as compared to female raters on all four agency dimensions. However, comparisons of the agency dimensions in ratings of men in general were not uniform and deviated from the overall agency results. Although men in general were rated no differently by male and female raters on the instrumental competence, assertiveness, or independence dimensions, female as compared to male raters rated men in general higher in leadership competence (again see Figure 1).

#### Communality

A 2 (rater gender: male, female) × 2 (target group: men in general, women in general) ANOVA of the overall communality ratings indicated only a main effect for target group, *F*(1,418) = 88.68, *p* < 0.001, $\eta_{p}^{2}$ = 0.18. The 2 × 2 × 3 mixed-model ANOVA (see Table 3), including the three communality dimensions as a within-subject factor, indicated main effects for target group, rater gender, and communality dimension as well as significant interactions between target group and rater gender, between communality dimension and target group, between communality dimension and rater type, and a three-way interaction.

##### Differences in ratings of men in general and women in general

LSD comparisons (see Table 4) for overall communality indicated that men in general were rated lower in communality than women in general by both male and female raters. In line with this overall finding, results of the LSD comparisons indicated that both female and male raters rated men in general as lower than women in general on all three communality dimensions: concern for others, sociability, and emotional sensitivity. Thus, using the overall measure yielded the same information as did the multidimensional measure.

##### Rater gender differences in target group characterizations

Additional LSD comparisons (again see Table 4) of the communality ratings indicated that both male and female raters rated men in general similarly in communality, but female raters rated women in general higher in communality than male raters did. LSD comparisons of male and female raters rating men in general using the three communality dimensions were aligned with the overall communality result: male and female raters did not differ in ratings of concern for others, sociability, or emotional sensitivity. However, when rating women in general, results of the LSD comparisons of male and female raters were aligned with the overall measure result for only two of the communality dimensions: Female raters rated women in general higher in concern for others and emotional sensitivity than male raters did. On the dimension of sociability, male and female raters did not differ in their ratings of women in general.

### Do Men and Women Differ in Their Self-Characterizations?

We used a one-way ANOVA to assess differences in men’s and women’s self-characterizations. We first analyzed the overall agency and communality ratings, and then conducted a mixed-model 2 × 4 ANOVA including the agency dimensions, and a 2 × 3 mixed-model ANOVA including the communality dimensions as a within-subject variable (see Table 5). We again followed up with LSD comparisons (see Table 6).

**Table 5:** 2 × 4 Agency ANOVA and 2 × 3 Communality ANOVA for self-ratings.

	2 × 4 Agency ANOVA	2 × 3 Communality ANOVA
Rater Gender Main Effect	*F*(1,204) = 1.93, *p* = 0.166, $\eta_{p}^{2}$ = 0.951	*F*(1,204) = 6.00, *p* = 0.015, $\eta_{p}^{2}$ = 0.03
Dimensions Main Effect	*F*(2,458) = 50.72, *p* < 0.001, $\eta_{p}^{2}$ = 0.20	*F*(2,391) = 23.25, *p* < 0.001, $\eta_{p}^{2}$ = 0.10
Dimensions ^∗^ Rater Gender	*F*(2,458) = 5.53, *p* = 0.003, $\eta_{p}^{2}$ = 0.03	*F*(2,391) = 3.50, *p* = 0.033, $\eta_{p}^{2}$ = 0.02


*Results are displayed for a 2 (self-rater gender: male, female) × 4 (agency dimensions: instrumental competence, leadership competence, assertiveness, independence) ANOVA and a 2 (self-rater gender: male, female) × 3 (communality dimensions: concern for others, sociability, emotional sensitivity) ANOVA.*

**Table 6:** Means (and standard deviations) and LSD results of self-ratings.

	Self-raters		LSD Self-rater Comparisons
		
	Men	Women	
**Overall Agency**	4.93 (1.10)	4.73 (1.02)	*p* = 0.215
Instrumental Competence	5.27 (1.38)	5.28 (1.20)	*p* = 0.961
Leadership Competence	4.92 (1.29)	4.45 (1.32)	*p* = 0.012
Assertiveness	4.56 (1.13)	4.14 (1.21)	*p* = 0.017
Independence	4.97 (1.30)	4.99 (1.24)	*p* = 0.910
**Overall Communality**	4.91 (1.20)	5.31 (1.15)	*p* = 0.013
Concern for Others	5.13 (1.47)	5.60 (1.35)	*p* = 0.009
Sociability	4.89 (1.25)	5.09 (1.23)	*p* = 0.223
Emotional Sensitivity	4.64 (1.28)	5.20 (1.21)	*p* = 0.002


*Means (and standard deviations) of male and female self-raters for all scales as well as LSD comparisons. Ratings were given on a 7-point scale from 1 “not at all” to 7 “very much”.*

#### Agency

**FIGURE 2:**
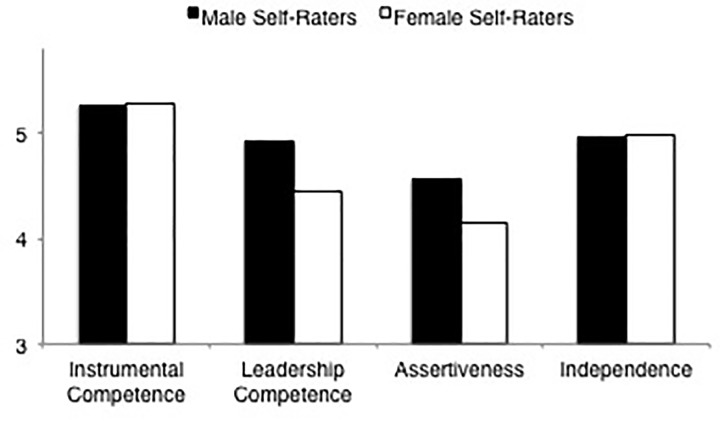
Ratings of agency dimensions (instrumental competence, leadership competence, assertiveness, independence) by male and female self-raters.

ANOVA results of the self-ratings of male and female raters on the overall measure of agency indicated no significant effect for rater gender, *F*(1,204) = 1.67, *p* = 0.198, $\eta_{p}^{2}$ = 0.01. However, results of the 2 × 4 mixed model ANOVA, with agency dimensions as the within-subject factor, indicated a main effect for agency dimension and an interaction between agency dimension and rater gender, suggesting that self-ratings differed depending on the agency dimension in question (see Table 5). LSD comparisons (see Table 6) of overall agency showed that, as was indicated by the non-significant gender main effects, women rated themselves as equally agentic as men. Yet, the results for the analyses including the four agency dimensions indicated that only findings for instrumental competence and independence were consisent with the pattern of results for the overall agency ratings (there were no differences in the self-ratings of female and male raters). There were, however, significant differences in ratings of leadership competence and in ratings of assertiveness. For both of these dimensions of agency, women rated themselves lower than men did (see Figure 2).

#### Communality

**FIGURE 3:**
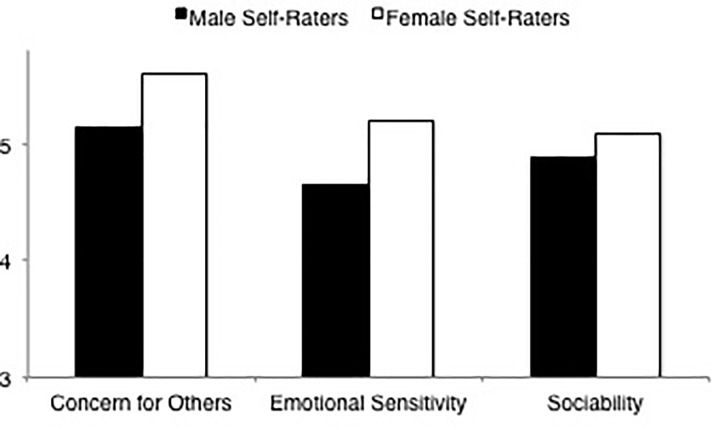
Ratings of communality dimensions (concern for others, emotional sensitivity, sociability) by male and female self-raters.

Results of the ANOVA of the self-ratings of male and female raters indicated a rater gender main effect, *F*(1,204) = 5.42, *p* = 0.021, $\eta_{p}^{2}$ = 0.03. Results of a 2 × 3 mixed-model ANOVA (again see Table 5) with communality dimension as the within-subjects factor, indicated significant main effects for rater gender and communality dimensions. LSD comparisons (again see Table 6), in line with the main effect for rater gender, indicated that men rated themselves lower on overall communality than women. LSD comparisons on the dimension scales indicated that, consistent with the overall communality results, men rated themselves as less concerned for others and less emotionally sensitive than women. However, in contrast to the results for overall communality, there was no difference in how men and women characterized themselves in terms of sociability (see Figure 3).

### Do Men’s and Women’s Self-Characterizations Differ From Their Characterizations of Their Gender Groups?

We used a 2 × 2 ANOVA, with rater gender (male, female) and target group (self, men in general when rater was male or women in general when rater was female) to assess differences in men’s and women’s self characterizations and same-sex others‘ characterizations of their gender groups. We first analyzed the overall agency and communality ratings, and then again conducted a 2 × 2 × 4 mixed-model ANOVA including our agency dimensions, and a 2 × 2 × 3 mixed-model ANOVA including our communality dimensions (see Table 7) and once more followed up with LSD comparisons (see Table 8).

**Table 7:** 2 × 2 × 4 Agency ANOVA and 2 × 2 × 3 Communality ANOVA for self-ratings versus target group ratings.

	2 × 2 × 4 Agency ANOVA	2 × 2 × 3 Communality ANOVA
Rater Gender Main Effect	*F*(1,397) = 1.76, *p* = 0.186, $\eta_{p}^{2}$ = 0.00	*F*(1,397) = 17.70, *p* < 0.001, $\eta_{p}^{2}$ = 0.04
Target Group Main Effect	*F*(1,397) = 0.03, *p* = 0.874, $\eta_{p}^{2}$ = 0.00	*F*(1,397) = 45.10, *p* < 0.001, $\eta_{p}^{2}$ = 0.10
Dimensions Main Effect	*F*(2,962) = 33.04, *p* < 0.001, $\eta_{p}^{2}$ = 0.08	*F*(2,785) = 11.63, *p* < 0.001, $\eta_{p}^{2}$ = 0.03
Rater Gender ^∗^ Target Group	*F*(1,397) = 3.11, *p* = 0.079, $\eta_{p}^{2}$ = 0.01	*F*(1,397) = 10.51, *p* = 0.001, $\eta_{p}^{2}$ = 0.03
Dimensions ^∗^ Rater Gender	*F*(2,962) = 31.32, *p* < 0.001, $\eta_{p}^{2}$ = 0.07	*F*(2,785) = 18.84, *p* < 0.001, $\eta_{p}^{2}$ = 0.05
Dimensions ^∗^ Target Group	*F*(2,962) = 12.32, *p* < 0.001, $\eta_{p}^{2}$ = 0.03	*F*(2,785) = 7.51, *p* = 0.001, $\eta_{p}^{2}$ = 0.02
Dimensions ^∗^ Rater Gender ^∗^ Target Group	*F*(2,962) = 4.42, *p* = 0.008, $\eta_{p}^{2}$ = 0.01	*F*(2,785) = 0.211, *p* = 0.646, $\eta_{p}^{2}$ = 0.00


*Results are displayed for a 2 (rater gender: male, female) × 2 (target group: self, men in general when the rater was male or women in general when the rater was female) × 4 (agency dimensions: instrumental competence, leadership competence, assertiveness, independence) ANOVA and a 2 (rater gender: male, female) × 2 (target group: self, men in general when the rater was male or women in general when the rater was female) × 3 (communality dimensions: concern for others, sociability, emotional sensitivity) ANOVA.*

**Table 8:** LSD comparisons of self-ratings versus target group ratings.

	Male Raters	Female Raters
**Overall Agency**	*p* = 0.046	*p* = 0.883
Instrumental Competence	*p* < 0.001	*p* = 0.038
Leadership Competence	*p* = 0.180	*p* = 0.004
Assertiveness	*p* = 0.353	*p* = 0.016
Independence	*p* = 0.039	*p* = 0.051
**Overall Communality**	*p* < 0.001	*p* = 0.367
Concern for Others	*p* < 0.001	*p* = 0.008
Sociability	*p* < 0.001	*p* = 0.943
Emotional Sensitivity	*p* = 0.001	*p* = 0.539


*Rating comparisons for male raters: Male self-raters versus male rater’s ratings of men in general; Rating comparisons for female raters: Female self-raters versus female rater’s ratings of women in general.*

#### Agency

The 2 × 2 ANOVA results for the overall agency measure indicated no significant main effect for rater gender, *F*(1,397) = 2.19, *p* = 0.139, $\eta_{p}^{2}$ = 0.00, or target group, *F*(1,397) = 0.013, *p* = 0.909, $\eta_{p}^{2}$ = 0.00, but a marginally signicant interaction between them, *F*(1,397) = 2.77, *p* = 0.097, $\eta_{p}^{2}$ = 0.01. The 2 × 2 × 4 mixed-model ANOVA including the agency dimensions as a within-subjects factor also indicated no significant main effects for rater gender or for target group and again a marginally significant interaction between them. It also indicated a significant main effect for agency dimension and significant interactions of dimension with both rater gender and target group, as well as a three-way interaction between rater gender, target group, and agency dimension (see Table 7).

##### Men’s self-ratings versus ratings of men in general

**FIGURE 4:**
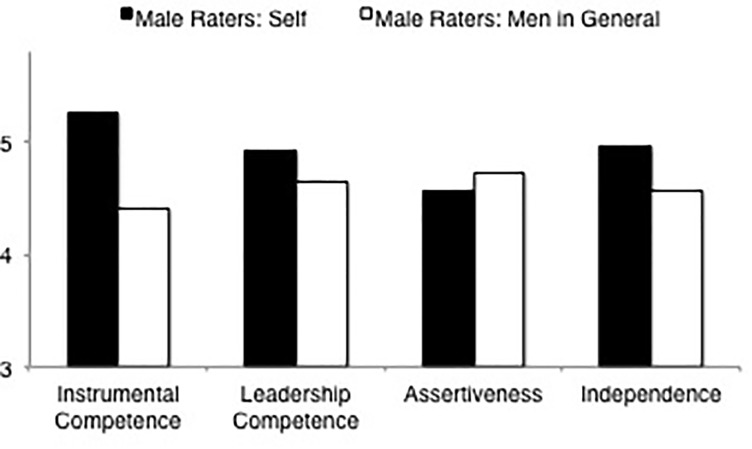
Ratings of agency dimensions (instrumental competence, leadership competence, assertiveness, independence) by male raters rating self and men in general.

LSD comparisons (see Table 8, means and standard deviations are displayed in Tables 4, 6) of overall agency indicated that male raters rated themselves as more agentic than male raters rated men in general. Results for the agency dimensions were more varied: For the independence and instrumental competence dimensions results were in line with the overall agency result, but male raters rated themselves no differently in leadership competence or assertiveness than male raters rated men in general (see Figure 4).

##### Women’s self-ratings versus ratings of women in general

**FIGURE 5:**
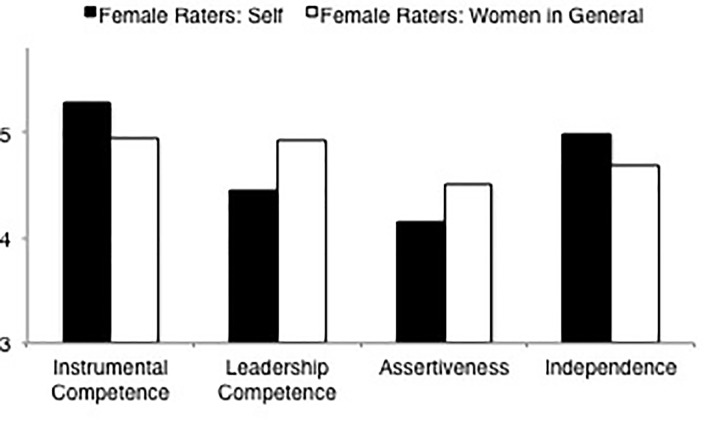
Ratings of agency dimensions (instrumental competence, leadership competence, assertiveness, independence) by female raters rating self and women in general.

LSD comparisons (see Table 8, means and standard deviations are displayed in Tables 4, 6) of the overall agency ratings indicated that female raters rated themselves no differently than female raters rated women in general. However, comparisons of the four agency dimensions depicted a different pattern. Although ratings of independence were in line with the overall agency result, female raters rated themselves higher in instrumental competence than female raters rated women in general. Most striking, however, were the differences in ratings on the leadership competence and assertiveness dimensions. In contrast to the findings for overall agency, in each of these cases female raters‘ ratings of themselves were significantly lower than female raters‘ ratings of women in general (see Figure 5). The differences in self-ratings of assertiveness and leadership competence marked the only instance in which there was a more negative characterization of self than of one’s gender group.

#### Communality

The 2 × 2 ANOVA results for the overall communality measure indicated a main effect for rater gender, *F*(1,397) = 19.03, *p* < 0.001, $\eta_{p}^{2}$ = 0.01, and target group, *F*(1,397) = 42.92, *p* < 0.001, $\eta_{p}^{2}$ = 0.10 as well as a significant interaction, *F*(1,397) = 10.51, *p* = 0.001, $\eta_{p}^{2}$ = 0.03. The 2 × 2 × 3 mixed-model ANOVA including the communality dimensions as a within-subjects factor indicated significant main effects for rater gender, for target group, and communality dimension as well as a significant interaction between rater gender and target group, between rater gender and communality dimension, and between target group and communality dimension (see Table 7).

##### Men’s self-ratings versus ratings of men in general

**FIGURE 6:**
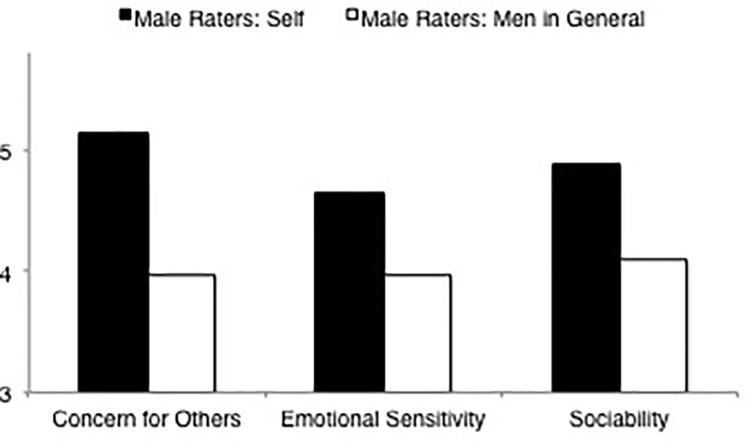
Ratings of communality dimensions (concern for others, emotional sensitivity, sociability) by male raters rating self and men in general.

LSD comparisons (see Table 8, means and standard deviations are displayed in Tables 4, 6) of overall communality indicated that male raters rated themselves as more communal than male raters rated men in general. LSD comparisons of the three communality dimension scales were consistent with the finding for overall communality. Male raters rated themselves significantly higher than male raters rated men in general in concern for others, sociability and emotional sensitivity (see Figure 6).

##### Women’s Self-Ratings Versus Ratings of Women in General

**FIGURE 7:**
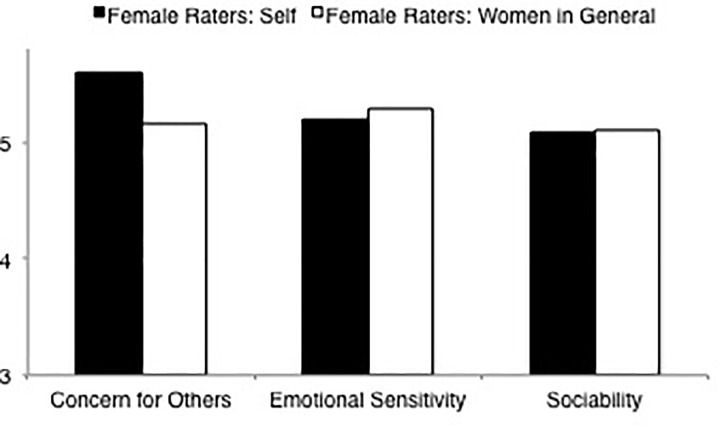
Ratings of communality dimensions (concern for others, emotional sensitivity, sociability) by female raters rating self and women in general.

LSD comparisons (see Table 8, means and standard deviations are displayed in Tables 4, 6) of the overall communality ratings indicated that there was no difference in how female raters rated themselves and how female raters rated women in general. LSD comparisons for sociability and emotional sensitivity were consistent with this finding. However, female raters rated themselves higher in concern for others than they rated women in general (see Figure 7).

## Discussion

It was the objective of this research to investigate gender stereotyping of others and self. To do so, we aimed to take into account multiple dimensions of the agency and communality constructs. It was our contention that perceptions on some of these dimensions of agency and communality would differ from one another, and that there would be a benefit in viewing them separately. Our results support this idea. While there were overall findings for agency and communality, analyses of individual aspects of them were not always consistent with these findings. What often appeared to be a general effect when using the overall measures of agency and communality in fact proved to be more textured and differentiated when the multidimensional framework was used. These results support the idea that distinguishing between different agency and communality facets can offer a deeper, more nuanced understanding of gender stereotypes today. Indeed, some important information appears to get lost by only focusing on the overall constructs.

### Answers to Our Research Questions

#### Current Stereotypes

Our results clearly indicate that gender stereotypes persist. They also indicate that stereotypes about agency were more prevalent for male than for female raters. Specifically, male raters described women in general as lower in most aspects of agency than men in general, and also rated women in general lower on each of the agency dimensions than female raters did. Nonetheless, female raters were not stereotype-free with respect to agency: they described women in general as less assertive than men in general and rated men in general as more leadership competent than male raters did. These findings were masked by the overall measure of agency, which indicated no differences in agency ratings.

Stereotypes about communality also were strongly indicated by our data, but their strength did not tend to differ greatly between male and female raters. All participants rated women higher than men on the three communality dimensions.

#### Self-Stereotyping

Our results showed that men’s and women’s self-characterizations differed in line with gender stereotypes. Despite the overall agency measure indicating no difference in self-ratings of agency, the analyses incorporating dimensions of agency painted a different picture. Whereas there was no difference in the self-characterizations of men and women in instrumental competence or independence, women rated themselves lower than men in leadership competence and assertiveness. There also were differences in communality self-ratings. Though men tended to rate themselves as generally less communal than women did (as less concerned for others and less emotionally sensitive), their ratings of sociability did not differ from women’s.

#### Self-Characterizations Versus Characterizations of One’s Gender Group

Self-characterizations were often found to differ from characterizations of one’s gender group. Male raters rated themselves as higher in independence and instrumental competence, but no different in assertiveness or leadership competence than they rated men in general. Female raters rated themselves higher in instrumental competence but lower in assertiveness and leadership competence than they rated women in general. These findings are at odds with the results of the overall agency ratings, which imply that male raters consistently rated themselves higher in agency, and that female raters consistently rated themselves no differently than they rated their gender group.

There also were differences between self-ratings and characterizations of one’s gender group on the communality dimensions. While female raters only rated themselves higher than they rated women in general in concern for others, male raters rated themselves as higher than they rated men in general on all three dimensions of communality.

### Implications

What does our analysis of current stereotypes tell us? On the one hand, our results indicate that despite dramatic societal changes many aspects of traditional gender stereotypes endure. Both male and female respondents viewed men in general as being more assertive than women in general, and also viewed women in general as more concerned about others, sociable and emotionally sensitive than men in general. On the other hand, our results indicate important departures from traditional views. This can be seen in the findings that unlike male respondents, female respondents indicated no gender deficit in how independent or how competent in leadership they perceived other women to be.

Self-descriptions also tended to conform to traditional gender stereotypes, with men describing themselves as more assertive and more competent in leadership than women did, and women describing themselves as more concerned about others and more emotional than men did. However, there were aspects of agency and communality for which self-characterizations of men and women did not differ. Women’s self-ratings of independence and instrumental competence were as high as men’s self-ratings, and men’s self-ratings of sociability were as high as women’s self-ratings. Together with the findings about characterizations of men and women in general, these results attest not only to the possible changing face of stereotypes, but also highlight the importance of considering specific dimensions of both agency and communality in stereotype assessment.

It should be noted that our results suggest a greater differentiation between the multidimensional results for agency characterizations than for communality characterizations. That is, the multidmenstional results more often aligned with the results of the overall measure when the focus of measurement was communality than when it was agency. It is not clear at this point whether this is because of the particular items included in our scales or because communality is a more coherent construct. But, based on our results, it would appear that the use of a multidimensional framework is of particular value when the measurement of agency is the focus – something that should be noted by those involved in studying stereotype assessment and change.

### Competence Perceptions

The lack of similarity in the pattern of results for the two competence dimensions (instrumental competence and leadership competence) is interesting. Although there were differences in ratings on the leadership competence dimension, ratings on the instrumental competence dimension did not differ when comparing ratings of men and women in general or when comparing male and female raters’ self-characterizations. It thus appears that there is an aspect of competence on which women are rated as highly as men – the wherewithal to get the work done. However, caution is urged in interpreting this finding. The attributes comprising the instrumental competence scale can be seen as indicative of conscientiousness and willingness to work hard, attributes often associated with women as well as men. Thus there is a question about whether instrumental competence is really a component of the agency construct, a question also prompted by its pattern of correlations with the other dependent measure scales (see also Carrier et al., 2014).

The leadership competence ratings paint a different picture. The consistent perception by men that leadership competence was more prevalent in men than in women suggests that, at least as far as men are concerned, women still are not seen as “having what it takes” to adequately handle traditionally male roles and positions. Whatever the interpretation, however, the different pattern of results found for these two scales indicates that we as researchers have to be very precise in designating what we are measuring and how we are measuring it. It also indicates that we have to keep close to the construct we actually have measured when drawing conclusions from our data.

### Women and Contemporary Gender Stereotypes

Our results show that women do not entirely embrace the stereotypic view of women as less agentic than men. They did not make distinctions between men and women in general when rating their independence and instrumental competence, nor were their self-ratings on the independence and instrumental competence scales lower than the self-ratings made by men. These findings are noteworthy: one of the key aspects of agency is independence, and it appears that women do not see themselves or other women to be lacking it more than men. Women also did not make distinctions between men and women in general when rating their leadership competence, another key component of agency. These findings suggest that, for modern day women, some important aspects of the agency stereotype no longer apply.

However, our results suggest that women have not moved as far along as one would hope in separating themselves from gender stereotypic constraints. In particular, their self-perceptions of assertiveness and leadership competence – dimensions of agency associated with social power – do not seem to deviate from traditional gender conceptions. Our findings indicate that women not only characterized themselves as less assertive and less competent in leadership than men characterized themselves, but they also described themselves significantly more negatively on these two scales than they described women in general. This means that women rated themselves as more deficient in several central aspects of agency than they rated women as a group, adhering more strongly to traditional gender stereotypes when describing themselves than when describing others. These results seem inconsistent with attribution theory (Jones and Nisbett, 1987) and construal level theory (Trope and Liberman, 2010), and challenge the idea that because people differentiate more when viewing themselves as compared to others they are less apt to use stereotypes in self-description. They also raise questions about differences in aspects of agency that do and do not involve power relations. These findings are in need of further exploration.

### Men and Contemporary Gender Stereotypes

Our results indicate that men continue to accept the stereotyped conception of men lacking communal qualities. They, along with women, rated men in general lower than women in general on all three communality dimensions. It therefore is particularly interesting that in their self-ratings on one dimension of communality – sociability – they did not differ from women. This finding suggests that men conceive of sociability differently when they characterize themselves than when they charcterize others. Other research suggests that whereas women are more social than men in close relationships, men are more social than women in group contexts (Baumeister and Sommer, 1997; Gabriel and Gardner, 1999). Thus, men might have rated themselves as equally sociable as women rated themselves, but for a different reason: because they conceptualized sociability with regard to their groups (rather than close relationships). If so, then clarification is needed about why this potentially different conception of sociability takes hold for men only when they characterize themselves.

Furtherore, it is of note that when comparing themselves with men in general, men’s ratings of themselves were significantly higher on all communal dimensions. This finding suggests that although they strongly adhere to traditional stereotypes in their characterizations of men as a group, there is a tendency for men to be less stereotype-bound when they characterize themselves. It also suggests that they are more self-aggrandizing when rating themselves than when rating other men – ascribing to themselves more of the “wonderful” traits traditionally associated with women (Eagly and Mladinic, 1989). This result contrasts with that found for women, for whom traditional gender stereotypes often appeared to exert more influence in self-characterizations than in characterizations of others, even when the result was self-deprecating rather than self-enhancing. Why there are differences in discrepancies in self-ratings versus other-ratings of women and men raises interesting questions for future research – questions about whether these differential effects are due to the gender of the rater or to the nature of the particular descriptors involved.

### Limitations

Our results indicate that breaking down agency and communality into dimensions was often of benefit when assessing stereotyped perceptions. Though many of our scales were highly correlated, the confirmatory factor analyses provided support that they were distinct facets. Our choice to analyze the scales separately despite high correlations is in line with other researchers, who argue that doing so can enhance results interpretation (Luthar, 1996; Tabachnik and Fidell, 2007). However, we do not claim that the dimensions we derived are the only way to differentiate among the elements of communality and agency, nor do we claim that our scales are the best way to measure them. Indeed, we chose a top–down procedure, using expert judges to derive our scales. This had the advantage that the judges knew about gender research and could effectively represent the literature on gender stereotypes. Nevertheless, if non-experts had done the initial sorting, they may have come to different conclusions about the number or content of items in the different scales or may have generated different scales altogether, ones that perhaps would have been more representative of everyday categories that are consensual in our culture.

Furthermore, our scale construction may have been constrained because our initial pool of items relied exclusively on existing items from past scales, which, although broadly selected, may have been limited by particular ways of thinking about stereotypes. Recent findings by Abele et al. (2016), for example, included a morality facet in their breakdown of communality, and found it to be a robust facet of communality in ratings within and between a large number of countries in both Eastern and Western cultures. We, however, did not include many items that measured morality in our original list of attributes. Whereas we scoured the gender stereotyping literature focused on social perception to compile the most frequently used items for our initial item pool, Abele and colleagues went through a similar process, but with literature focused primarily on self-perception. Items focusing on the morality component of communality should no doubt be incorporated in future research. In addition, there might also be additional items relating to other facets of agency, such as a cognitive agency facet (e.g., being rational). Moreover, and more generally, a process by which the attributes comprising the scales are generated in a free-form manner and the categorization tasks are performed by a broad-ranging set of judges would serve as a check on our measures and provide guidance about how to modify and improve them.

There are other methodological limitations that are suggestive of follow-up research. We found no differences as a result of the rater’s age and education, attesting to the generality of the effects we uncovered, but there no doubt are other possible moderating factors to be explored, such as race and socio-economic level. Moreover, although we were able to tap into a wide-ranging population, it is important to replicate our study with a more representative U.S. sample to assess the full scope of our findings. In addition, our study was restricted to a sample of U.S. citizens, and it would be interesting to replicate this research with samples that are not exclusively from the U.S. Such cross-cultural replications would help not only to assess generalizability to other cultures, but also to assess the extent to which the nature and degree of change in social roles influences the way people currently conceive of men and women, and men and women conceive of themselves. Finally, it would be useful to conduct research using our measure to describe more differentiated targets to determine whether our results would be similar or different when intersectionality is taken into account and when particular subtypes of women and men are the focus.

### Going Forward

Our findings stimulate several questions for future research. Not only would it be useful to further investigate the competence component of agency, clarifying what it does and does not entail, but also to consider another aspect of competence that has recently been identified as being strongly male gender-typed – intellectual brilliance (Leslie et al., 2015). Exploring the effects of the apparently contradictory view women have of themselves in terms of agency (self-views of their independence and instrumental competence versus self-views of their assertiveness and leadership competence) on women’s attitudes and behavior in a variety of spheres also would be valuable. In addition, it would be advantageous to determine whether the greater communality men ascribe to themselves than to other men reflects actual beliefs or is merely self-enhancing, and if it has implications for men’s approach to traditionally female roles and positions.

Finally, it is important that in future research attempts are made to demonstrate the usefulness of distinguishing among the dimensions of agency and communality we have identified, and to do so for both self and other characterizations. While for some research questions an overall agency and overall communality measure will likely be sufficient, there no doubt are instances in which finer distinctions will be beneficial. It is possible, for example, that different dimensions of gender stereotypes are more strongly associated with selection decisions, performance evaluations, or reward distributions. Indeed, other researchers have already begun to demonstrate the value of considering distinct facets of agency in assessing gender differences in leader evaluations, but with a less differentiated set of dimensions including only self-reliance and dominance (Schaumberg and Flynn, 2017). It also is possible that different dimensions of self-stereotypes are more strongly associated with career aspirations and choices, or support for gender-related organizational policies. Demonstrating that different dimensions of agency and communality predict different outcomes would add support to our multidimensional framework. In addition to increasing our understanding, such discoveries could provide valuable information about leverage points for intervention to ease the negative consequences of gender stereotyping and the bias they promote.

## Conclusion

In this study we have demonstrated the value of subdividing the agency and communality construct in the study of gender stereotypes, and shown that making global statements about agency and communality runs the risk of distorting rather than clarifying our understanding.

Our goal with this paper was to further the conversation in the field about different aspects of both agency and communality and their potentially different effects on self and other characterizations. An underlying theme is that we may be losing information by generalizing to two super constructs and not attending to their components. Our findings demonstrate the complexity of the agency and communality constructs and the potential benefits of thinking about them with greater specificity. This can have consequences not only for understanding stereotypes and gender bias, but also for intervention and change efforts.

What are the implications of our findings for understanding the persistence of gender inequality? Although the results signal easing in some dimensions of traditional gender stereotypes, they make clear that in many ways they persist. Of particular importance is men’s unrelenting image of women as deficient in attributes considered to be essential for success in many traditionally male fields – an image that forms the basis of gender bias in many evaluative decisions. But women are not exempt from the influence of gender stereotypes; even though they view women as equal to men in several key agentic qualities, they see themselves as more deficient than men do in both leadership competence and assertiveness, and more deficient in these agency dimensions than women in general. These findings, which result from consideration of multiple aspects of the agency construct, augur ill for the tempering of women’s tendency to limit their opportunities. Evidently we still have a way to go before all the components of traditional gender stereotypes fully dissipate and recede, allowing men and women to be judged, and to judge themselves, on the basis of their merits, not their gender.

## Ethics Statement

This study was carried out in accordance with the recommendations of the Institutional Review Board, University Committee on Activities Involving Human Subjects, New York University. The protocol was approved by the University Committee on Activities Involving Human Subjects, New York University.

## Author Contributions

All authors listed have made a substantial, direct and intellectual contribution to the work, and approved it for publication.

## Conflict of Interest Statement

The authors declare that the research was conducted in the absence of any commercial or financial relationships that could be construed as a potential conflict of interest.

## Footnotes

1 The median age of the U.S. population is 37.9 years (United States Census Bureau, 2017c); Levels of education of the U.S. population 25 years and older in 2017: 39.2% did not attend college, 16.3% had some college, 31.6% had graduated college, 12.9% have graduate degrees (United States Census Bureau, 2017a); Race/ethnicity percentages in the general U.S. population are as follows: 60.7% White, 18.1% Hispanic, 13.4% African American, 5.8% Asian, 2% other (United States Census Bureau, 2017b).

2 The attributes in the inventory included the communal and agentic attributes of interest as well as a group of attributes measuring other constructs that were included for exploratory purposes but not used in this study.

3 The relatively large RMSEA is likely due to violation of multivariate normality assumptions (joint multivariate kurtosis = 76.55 with a critical ratio of 55.30). The most important implication of non-normality is that chi-square values are inflated, whereas parameter estimates are still fairly accurate (Kline, 2011).

## Appendix

**Table A:** List of agentic attributes measured.

Agentic Attributes	
Able to Separate Feelings from Ideas	Independent
Achievement-Oriented	Intelligent
Active	Leadership Ability
Ambitious	Logical
Analytical	Objective
Assertive	Organized
Authoritative	Persistent
Bold	Productive
Competent	Relaxed
Competitive	Reliable
Conscientious	Risk-Taking
Consistent	Self-Confident
Decisive	Self-Controlled
Desires Responsibility	Self-Reliant
Direct	Skilled In Business Matters
Dominant	Sophisticated
Effective	Speedy Recovery From Emotional Disturbance
Emotionally Stable	Stands Up Under Pressure
Feelings Not Easily Hurt	Steady
Firm	Strong
Forceful	Task-Oriented
High Need For Power	Vigorous
High Self-Regard	Well-Informed

**Table B:** List of communal attributes measured.

Communal Attributes	
Affectionate	Likeable
Aware of Others Feelings	Modest
Cheerful	Neat
Collaborative	People-Oriented
Communicative	Relationship-Oriented
Compassionate	Sensitive
Emotional	Sentimental
Generous	Sincere
Gentle	Sociable
Good Natured	Sympathetic
Helpful	Talkative
Humanitarian Values	Tender
Intuitive	Understanding
Kind	Warm
